# 

**Published:** 1881-04-20

**Authors:** 


### *EDITORIAL NOTICES.*

*Buffalo Lithia Springs.*—We invite attention to the advertisement of the above celebrated Springs in this issue of our Journal.

*The International Medical Congress* will assemble in London on Wednesday, August 3, 1881. It is supposed that it will be the largest medical body which ever before assembled in the world.

*Reed & Carnrick.*—See their advertisement in this journal. Their malt combinations are excellent and exceedingly convenient to the practitioner, furnishing a variety adapted to almost every indication.

*Liebig & Co.'s Coca Beef Tonic.*—See the advertisements of this establishment. We previously alluded to the excellency of their preparations. The enterprising house of J. L. Berg & Co., 60 Maiden Lane, New York, are the sole agents in this country for the sale of these goods.

### *OFFICERS ELECT OF THE SOUTHERN MEDICAL COLLEGE*

At a late meeting of the Faculty Dr. R. C. Word tendered his resignation as Dean of the Southern Medical College upon the ground that its duties in connection with his editorial and professional labors had proven too great a tax upon his health. Whereupon the Faculty passed the following complimentary resolution:

*Resolved.* That the thanks of the Faculty are due, and are hereby tendered to Dr. R. C. Word, Dean, for his faithful and conscientious management of the office of Dean of the College, and that we give him the plaudit of "Well done good and faithful servant."

The officers of the Faculty in this Institution are elected annually. The following are chosen for the ensuing collegiate year, ending March, 1882, viz:

THOMAS S. POWELL, President.

R. C. WORD, Vice-President.

W. P. NICOLSON, Dean.

J. J. TOON, Treasurer.

*RECEIPTED.*

[Receipts not acknowledged privately are entered here.]

1880—Drs J E Pope, W M Garrard, L P Hamner, Thos A Cook, W A Cusick, D S Williams, C N Howard, 1870–R B Stapleton. 1879, '80.–R W Rea.

1881—Drs R L Seale, L S Brownlee, M V Miller, T E Morris, T C Davis, Jno Hardman, E F Sumerall, O M Doyle, James Ray, J A Siddons, C W Keller, T P Davis, J J Groover, T F Houston, D R Fox, H E Hurst, Jno Lee, J B Fonville, J R Muse.

